# The Influence of Pd and Zr Co-Doping on the Microstructure and Oxidation Resistance of Aluminide Coatings on the CMSX-4 Nickel Superalloy

**DOI:** 10.3390/ma14247579

**Published:** 2021-12-09

**Authors:** Jolanta Romanowska, Jerzy Morgiel, Maryana Zagula-Yavorska

**Affiliations:** 1 Department of Materials Science, Faculty of Mechanical Engineering and Aeronautics, Rzeszów University of Technology, 2 W. Pola Street, 35-959 Rzeszów, Poland; jroman@prz.edu.pl; 2 Institute of Metallurgy and Materials Science PAS, 25 Reymonta Street, 30-059 Kraków, Poland; j.morgiel@imim.pl

**Keywords:** nickel superalloys, aluminide coatings, co-doping, oxidation resistance

## Abstract

Pd + Zr co-doped aluminide coatings were deposited on the CMSX-4 nickel superalloy, widely used in the aircraft industry, in order to investigate their microstructure and improvement of oxidation resistance. Palladium was deposited by the electrochemical method, whereas zirconium and aluminum by the chemical vapor deposition (CVD) method. Coatings consist of two zones: the additive and the interdiffusion one. The additive zone contains β–(Ni,Pd)Al phase with some zirconium-rich precipitates close to the coating’s surface, whereas the interdiffusion zone consists of the same β–(Ni,Pd)Al phase with inclusions of refractory elements that diffused from the substrate, so called topologically closed-packed phases. Palladium dissolves in the β–NiAl phase and β–(Ni,Pd)Al phase is being formed. Pd + Zr co-doping improved the oxidation resistance of analysed coatings better than Pd mono-doping. Mechanisms responsible for this phenomenon and the synergistic effect of palladium and zirconium are discussed.

## 1. Introduction

High temperature and aggressive environment are both detrimental factors causing damage and shortening the lifetime of machine parts like turbine blades located in hot section of aircraft engines. That is why so many efforts are undertaken to protect these parts against hot corrosion and oxidation. Turbine blades made of Ni-based superalloys [1] are nowadays routinely protected by thermal barrier coatings (TBCs) [2] consisting of ceramic topcoats and bondcoats [3]. The top coating is usually an yttria-stabilized zirconia (YSZ) layer deposited by the EB-PVD method, producing numerous pores and pathways enabling high oxygen permeability. Therefore, the bondcoat, situated between the ceramic topcoat and the substrate, must be oxidation resistant. Moreover, it ensures top coating’s good adhesion and mitigates thermal mismatch stresses [4]. The most popular bondcoats are MCrAlY ones (M denotes Ni and/or Co) and aluminide coatings. The MCrAlY coatings are deposited by HVOF (high-velocity oxygen fuel spraying) [5], LPPS (low-pressure plasma spraying) [6], or APS (air plasma spraying) [7] methods.

The MCrAlY coating contains up to two phases: β–NiAl and γ–Ni. Each of these phases forms their own oxides [1]. Aluminide coatings are deposited by ‘out of pack’, pack cementation, and chemical vapor deposition (CVD) methods [2]. During oxidation, the single phase β–NiAl coating forms a homogenous Al_2_O_3_ layer [8]. Aluminide coatings are degraded by the loss of aluminum due to oxidation of the coatings’ surface forming aluminum oxide and interdiffusion between coatings and substrates [9]. An improvement in this area could be achieved only through slowing down the oxide scale growth rate and securing its better adherence [10]. The modification of aluminide coatings by noble metals, like palladium [11] or platinum [12], reduces the oxide scale growth rate and improves their adherence to the substrate, as well as delays β to γ′ phase transformation. The addition of noble metals significantly slows down the diffusion of refractory alloying elements from the substrate eliminating formation of chromium-rich inclusions in the outer layer of the coating [4]. Main phases in modified coatings deposited on CMSX-4 and Inconel 713 LC nickel-based superalloys are: (Ni,Pd)Al or (Ni,Pt)Al, respectively. Inclusions of M_23_C_6_ are found in Inconel 713 LC, whereas σ and µ phases in CMSX-4 superalloy [13].

Reactive elements, such as zirconium, hafnium, yttrium, or cerium, are also applied as modificators in aluminide coatings, as they also improve adhesion and reduce the oxide scale growth rate [14]. This phenomenon is attributed to the segregation of reactive elements on boundaries of oxide grains [15]. It slows down oxygen ions diffusion through the scale and prevents the formation of voids [16]. Zirconium improves the oxidation resistance of aluminide coatings [14], accelerates Al_2_O_3_ formation, and inhibits outward Al diffusion [17]. Moreover, zirconium increases the corrosion resistance and plasticity of the β–NiAl phase [18] and creep resistance, lowering at the same time internal stresses [19]. ‘Co-doping’, i.e., implementing more than one modificator to a coating, is becoming more and more popular [20] due to better oxidation resistance than that achieved in single-doped ones. When ions of two dopants segregate along grain boundaries in an oxide scale, their interaction with Al ions is stronger than for one dopant’s ions, i.e., a synergistic effect is observed [21]. The performed experiments confirmed that Pt + Zr co-doped aluminide coatings have better oxidation resistance and adhesion and lower surface roughness than any single-doped ones [22]. Moreover Zr-rich oxide pegs improve phase stabilities in the coating and reduce oxide scale growth rate [23]. The Pd + Hf is another promising co-doping pair. Pd + Hf co-doped aluminide coatings on CMSX-4 nickel superalloy have a two-phase structure, i.e., they are built of the β–(Ni,Pd)Al phase and the Hf-doped β–(Ni,Pd)Al phase with inclusions of µ and σ phases. Pd is distributed in both phases, whereas Hf forms inclusions. The oxidation resistance of this co-doped coating is better than the single-doped one [24]. As the creep resistance of CMSX-4 nickel superalloy is good, it is widely used in the aircraft industry, e.g., on turbine blades that are exposed to high temperatures and aggressive environments in the hot section of aircraft engines. Unfortunately, the oxidation resistance of this alloy is rather poor, and therefore finding a protective coating that would improve oxidation resistance of CMXS-4 superalloys is very important, because this way the lifetime of aircraft engines could be prolonged [4]. So far, the authors of this paper have analyzed the influence of several modifiers, e.g., Pd [4] or Pd + Hf [24], and observed that the modifiers mentioned above improve the oxidation resistance of coatings and that co-doping is more effective than mono-doping.

These promising results encouraged the authors of this paper to continue research on co-doping. Therefore, the research presented in this paper focuses on the analysis of the synergistic effect of palladium and zirconium, an as-yet untried pair of modifiers, and their role in aluminide coatings deposited on the CMSX-4 superalloy.

## 2. Experimental Procedure

Co-doped aluminide coatings were deposited on the CMSX-4 superalloy of the following nominal composition: Ni-61.5, Cr-6.5, Mo-0.6, Ta-6.5, Al-5.6, Ti-1, Co-9, W-6, He-0,1, Re-3 wt%. Samples were cut from the rod, polished, degreased ultrasonically in acetone, and etched [25]. Palladium layers were deposited by the electrochemical method in three steps (Table 1, Table 2 and Table 3):

1. Nickel Electroplating—t = 6 min, T = 20 °C, Current Density 3A dm^−2^ in the Bath of the following Chemical Composition [26].

**Table 1:** The chemical composition of the Ni plating bath.

Constituent	Concentration
Nickel(II) chloride (NiCl_2_∙6H_2_O)	240 g∙dm^−3^
Hydrochloric acid (35% wt.)	31 cm^3^∙dm^−3^
Deionised water	to 1 dm^3^

2. Acidic Palladium Strike Process—t = 90s, T = 40 °C, Current Density 5mAcm^−2^ in the Bath of the following Chemical Composition [26].

**Table 2:** The chemical composition of the Pd strike bath.

Constituent	Concentration
Palladium(II) chloride	1.6 g∙dm^−3^
1,2-diaminopropane	5.4 cm^3^∙dm^−3^
Glacial acetic acid	23.3 cm^3^∙dm^−3^
Sodium chloride	60 g∙dm^−3^
Deionised water	to 1 dm^3^

The well-adherent, 50-nm thick Pd layer was obtained.

3. Palladium Electrodeposition—t = 11 min, T = 55 °C, Current Density 10 mAcm^−2^ in the Bath of the following Chemical Composition [27].

**Table 3:** The chemical composition of the Pd plating bath.

Constituent	Concentration
Palladium(II) chloride	13.3 g∙dm^−3^
diethylenetriamine	16.2 cm^3^∙dm^−3^
Phosphate buffer	to 1 dm^3^

The 3 µm thick Pd layer was deposited.

Aluminide coatings were deposited on the CMSX-4 alloy with a palladium layer by the CVD method in the following steps:

1. Heating from room temperature up to 1050 °C
2. Aluminizing at 1050 °C for 30 min
3. Aluminizing and zirconizing at 1050 °C for 90 min
4. Aluminizing at 1050 °C for 240 min
5. Cooling to room temperature

**Figure 1:**
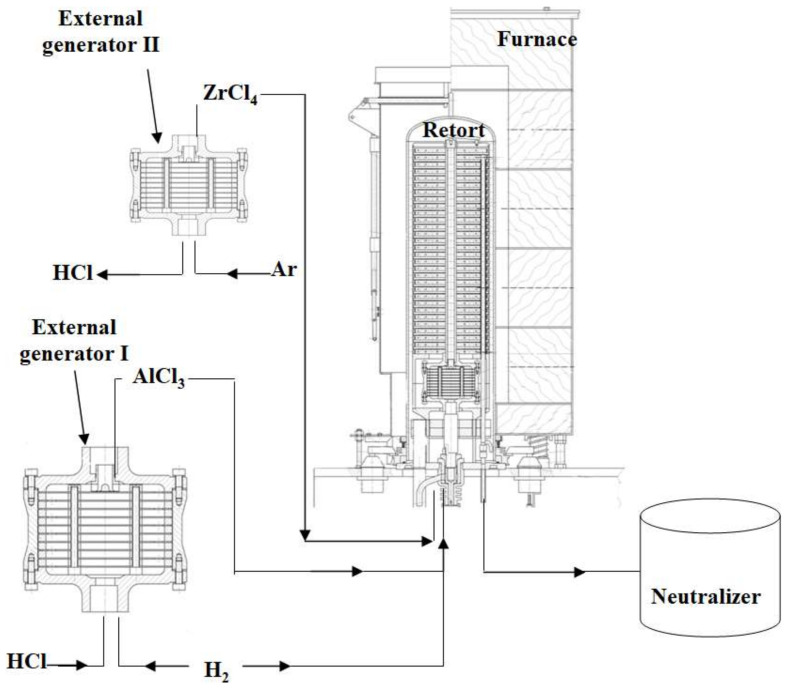
CVD equipment.

This process was carried out using the semi-industrial BPX-Pro325 equipment manufactured by IonBond Company (Olten, Switzerland) (Figure 1).

**Figure 2:**
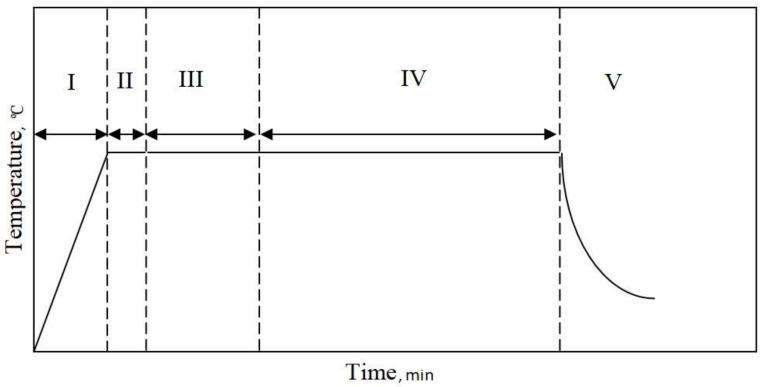
Temperature profile of the Zr + Al CVD deposition process.

Aluminium chloride vapor (AlCl_3_) was produced in the external generator I (Figure 1) with aluminium pellets by running HCl gas. Zirconium chloride vapor (ZrCl_4_) was produced in external generator II (Figure 1) with zirconium pellets by running HCl gas. Both precursors (AlCl_3_ and ZrCl_4_) were transported to the retort, in which samples with Pd layer were placed, and heated to 1050 °C. The AlCl_3_ vapor reacted more with the ZrCl_4_ vapour than with nickel at the temperature 1050 °C and grains of intermetallic phase NiAl(Zr) were formed. The temperature profile of the process is presented in Figure 2.

The coatings’ microstructure was analyzed using the scanning electron microscope (SEM) equipped with the energy dispersive spectroscope (EDS), electron backscattered diffraction (EBSD), and X-ray diffraction (XRD) methods. Parameters of the CVD process were carefully chosen on the basis of previous experiments [28]. Oxidation tests were performed at 1100 °C, a little bit higher than the operating temperature of coated parts, e.g., blades of an aircraft engine. The exposure time is long enough to notice the coating’s degradation. The atmosphere is the same in which coated blades operate.

## 3. Results and Discussion

### 3.1. Palladium and Zirconium Co-Doped Aluminide Coating

**Figure 3:**
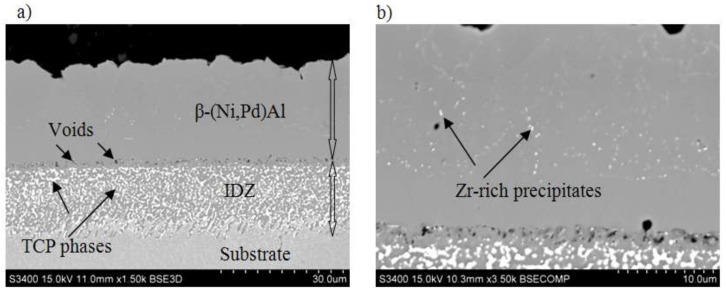
SEM-BSE microstructure on the cross-section of the palladium and zirconium co-doped aluminide coating (**a**) additive zone (**b**).

**Figure 4:**
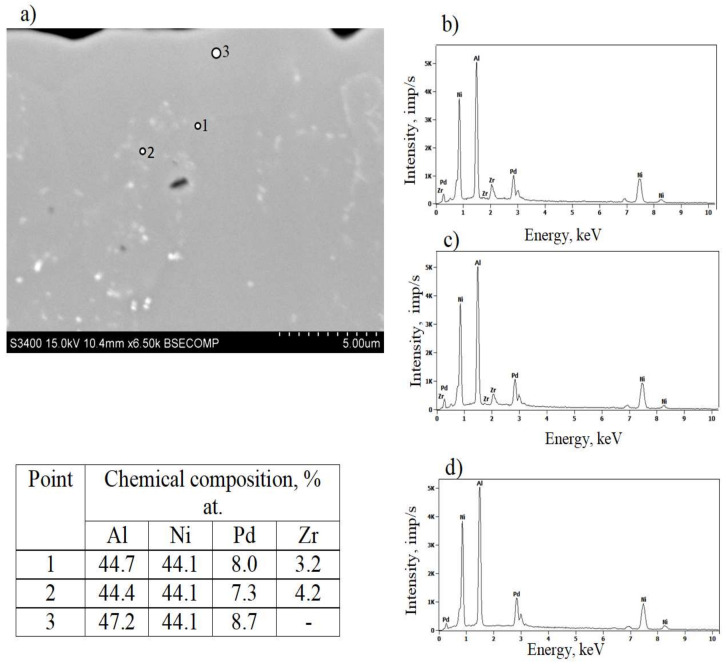
SEM microstructure on the cross-section of the additive zone of the palladium and zirconium co-doped aluminide coating (**a**) and EDS spectrum of point 1 (**b**), of point 2 (**c**) and of point 3 (**d**).

**Figure 5:**
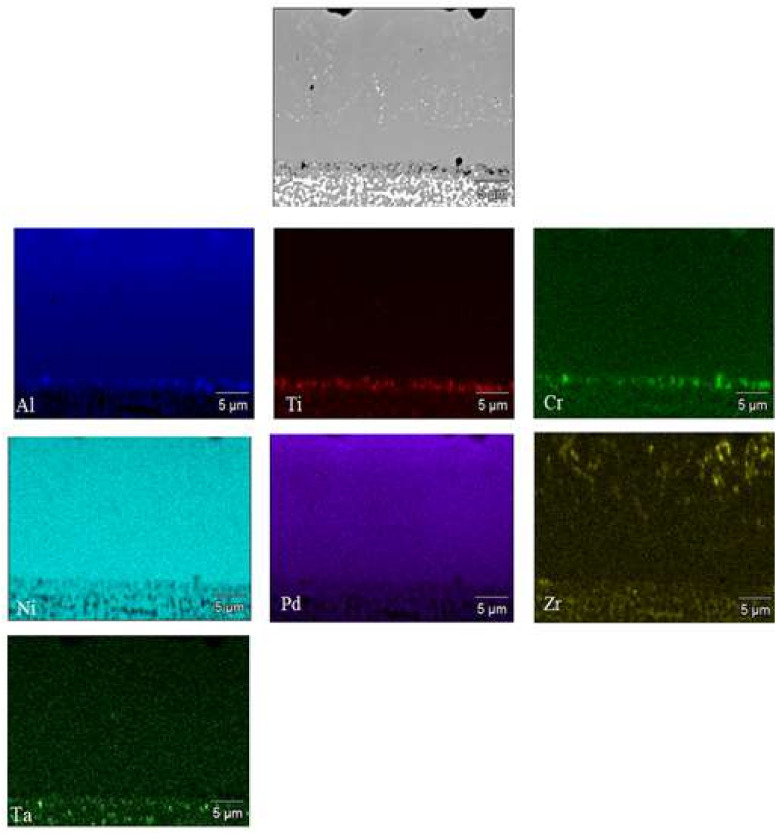
SEM micrograph of the additive zone with corresponding maps presenting distribution of Al, Ti, Cr, Ni, Pd, Zr and Ta.

Figure 3 shows a cross section of the coated specimen. The double-zone structure is visible. The outer, additive zone (Figure 3a) is 24 μm thick. Its chemical composition (Figure 4a, point 3), namely 47.2% at. Al, 44.1% at. Ni, and 8.7% at. Pd, corresponds to the β–(Ni,Pd)Al phase. The interdiffusion zone, situated below, is 16 μm thick (Figure 3a). Precise analysis of phase composition of the interdiffusion zone of the aluminide coating deposited on CMSX-4 nickel superalloy has been presented in references [24,29]. Its zone consists of the β–NiAl matrix and Topologically Closed-Packed (TCP) phases rich with refractory elements [29]. Palladium is dissolved in the β–NiAl phase and this way the β–(Ni,Pd)Al phase is being formed [24]. Moreover, a few voids are visible at the additive–interdiffusion zone (IDZ) interface (Figure 3a). A lot of bright globular Zr-rich precipitates (0.5 µm) are in the middle and upper part of the additive zone (Figure 3b and Figure 4a–c, Points 1–2). Aluminum, nickel, and palladium peaks (Figure 4d, Point 3) in the EDS spectrum of point 3 indicate the presence of the β–(Ni,Pd)Al phase. The formation of the β–(Ni,Pd)Al phase is confirmed by the uniform distribution of nickel, aluminium, and palladium in the additive layer (Figure 5). Zirconium is located in globular precipitates (Figure 5).

**Figure 6:**
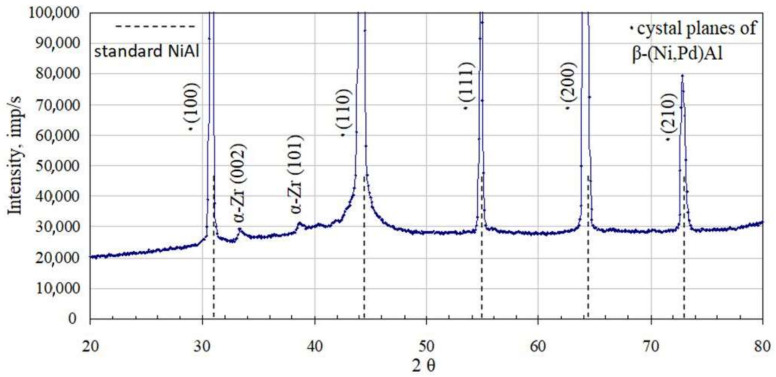
XRD diffraction pattern of the palladium and zirconium co-doped aluminide coating deposited on CMSX 4 superalloy.

**Figure 7:**
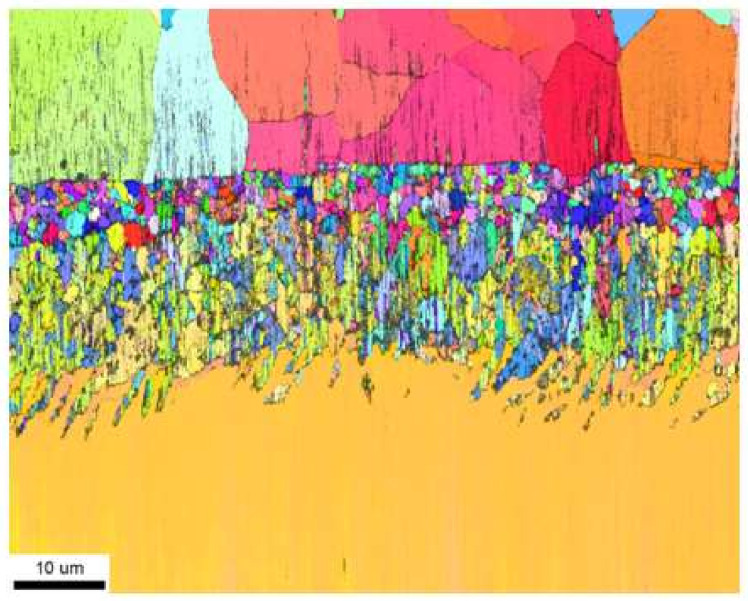
Map of crystallographic miss-orientation of crystallites in the palladium and zirconium co-doped aluminide coating.

**Figure 8:**
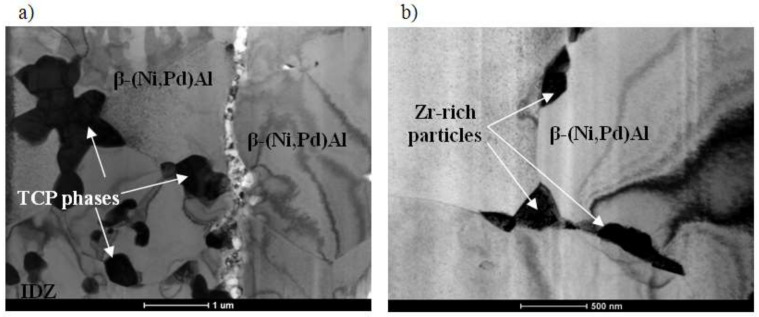
TEM microstructure of cross-section of the palladium and zirconium co-doped aluminide coating: interdiffusion/additive zone (**a**) and additive zone (**b**).

**Figure 9:**
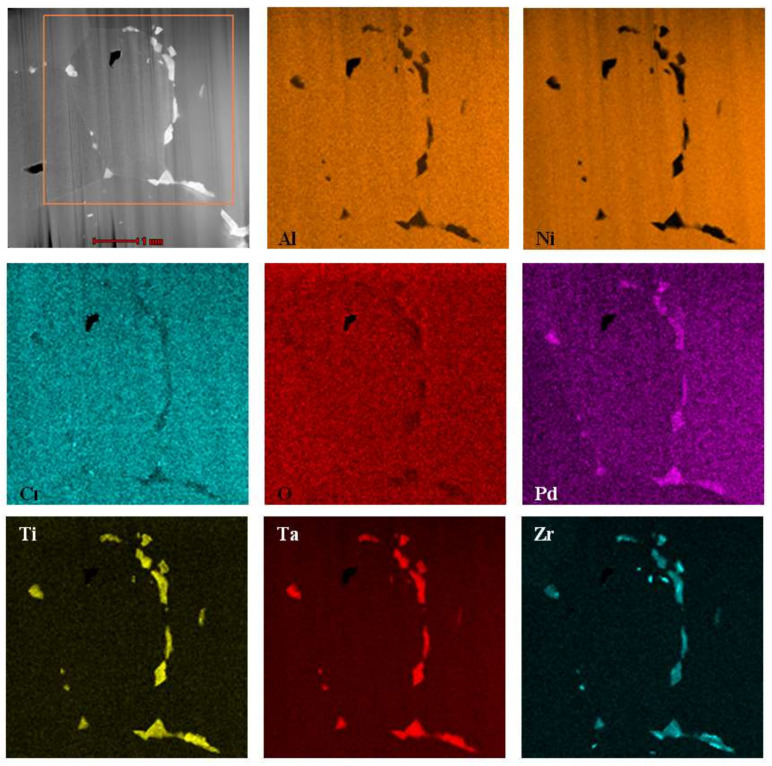
STEM-HAADF micrograph of Zr and Pd co-doped aluminide coating with maps presenting distribution of Al, Ni, Cr, O, Pd, Ti, Ta and Zr.

X-ray diffraction of palladium and zirconium co-doped aluminide coating (Figure 6) is shifted left to the non-modified one (dashed line). This proves that palladium enlarged the NiAl crystal lattice. Moreover, there are two small peaks of the α-Zr phase. It confirms successful Pd and Zr co-doping of the NiAl phase. Palladium formed the β–(Ni,Pd)Al phase, whereas zirconium precipitated as the α-Zr phase (hexagonal structure with cell parameters: a = 3.232 nm, c = 5.147 nm). The analysis of local crystal orientation performed by the EBSD method clearly confirmed 2-zones structure of the coating. The lower interdiffusion zone is filled with much coarser crystallites than the upper additive one (Figure 7). Inclusions in the interdiffusion zone prevented crystallites from coarsening. In Figure 8a,b, a great number of inclusions is visible. Zr rich inclusions (250 nm to 500 nm diameter) precipitate in the additive zone (Figure 8b and Figure 9), whereas TPC phases are in the interior and at grain boundaries in the interdiffusion zone (Figure 8b). The EDS spectrum of TCP phases of the aluminide coatings co-doped with Pd and Hf deposited on the CMSX-4 superalloy was obtained by TEM analysis [24]. This indicated that the TPC phases are built of Ti, Cr, Co, W, Ta, and Mo. The low solubility of alloying elements in the co-doped aluminide coating resulted in the precipitation of topologically closed-pack phases (μ and σ) in the interdiffusion zone [24].

The uniform distribution of nickel, aluminum, and palladium in the additive zone, analyzed by the TEM microscope (Figure 9), confirms palladium dissolution in the β–NiAl phase and formation of the β–(Ni,Pd)Al phase. The uniform distribution of nickel, aluminum, and palladium was also observed in the additive zone of Pd + Hf co-doped aluminide coatings on the CMSX-4 nickel superalloy [24]. Inclusions containing zirconium, tantalum, and titanium precipitated at grain boundaries. The precipitates located on the β–(Ni,Pd)Al phase grain boundaries of the additive zone are mostly composed of zirconium, tantalum, and titanium. According to Jiang et al. [30], a considerable amount of Ta and Zr segregate in inclusions between grain boundaries in the oxide scale. This phenomenon agrees with the dynamic-segregation theory [31]. However, the influence of Ta and Zr on the growth of oxide and phase precipitation remains unknown. Hafnium-rich precipitates locate at the additive and interdiffusion zone interface in the Pd + Hf co-doped aluminide coatings on CMSX-4 nickel superalloy [24]. This precipitate effectively acts as a diffusion barrier to block the inward diffusion of aluminum and the outward diffusion of substrate elements, thus oxidation resistance of the palladium and hafnium doped aluminide coating is better than only palladium modified one [24].

According to Lamesle et al. [32], the β–(Ni,Pd)Al phase has a B2 crystalline structure, like β–NiAl. Its formation is governed by Ni, Al, and Pd diffusion. Palladium diffusion dominates in the initial stage of the coating formation, and then the growth of the β-(Pd,Ni)Al phase is controlled by aluminium and nickel diffusion. Aluminium diffusion predominates in the outer layer, whereas nickel diffusion predominates in the part of the coating closer to the substrate [32]. The Pd_x_Ni_1−x_ + Al′ → (Pd_x_Ni_1−x_)Al reaction zone is situated at the crossover of these two fluxes. The interdiffusion zone is situated below with a great number of inclusions of refractory elements that diffused from the substrate. The additive zone, above, contains a relatively small number of zirconium-rich precipitates. Precipitates located along grain boundaries hinder further growth of the β–(Ni,Pd)Al phase and usually show a high tendency for coarsening. The density of inclusions in the interdiffusion zone is much higher than in the additive zone (Figure 3), which results in a much finer microstructure (Figure 7). Even as the inclusions in the additive zone are fewer than those in the interdiffusion one, and therefore less effective in restricting grain growth, they diminish the formation of voids on the surface [22].

### 3.2. Oxidation Behavior of Palladium versus Palladium and Zirconium Co-Doped Aluminide Coating

**Figure 10:**
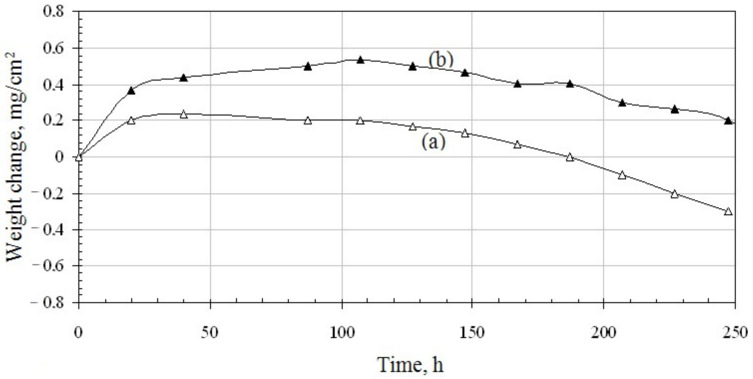
Weight change versus oxidation time of the palladium doped (**a**) and palladium and zirconium co-doped (**b**) aluminide coatings.

An oxidation resistance test was performed for two coated samples of the CMSX-4 nickel based superalloy. The aluminide coating on the first sample was palladium doped, whereas the aluminide coating in the other coating was palladium and zirconium co-doped. The mass change and the samples’ area were measured. The weighting accuracy was 0.0001 g and the samples’ area measurements’ accuracy was 0.001 cm. The measurement error was smaller than 0.01% and the mass change of the samples for the samples’ area was calculated and is presented in Figure 10.

Figure 10 presents change curves versus time of palladium doped and palladium and zirconium co-doped coatings. Weight uptakes of the palladium and zirconium co-doped coating are bigger, than of the palladium doped one. During the initial oxidation stage, so called incubation period (20 h), the weight gain of both coatings increases significantly (up to 0.2 mg/cm^2^ for palladium doped coating and up to 0.38 mg/cm^2^ for palladium and zirconium co-doped coating). In this incubation period, metastable cubic θ-Al_2_O_3_ is formed and next it transforms to the stable hexagonal α-Al_2_O_3_. θ-Al_2_O_3_ grows faster than α-Al_2_O_3_ and transformation takes place when α-Al_2_O_3_ nuclei have critical grain size. Large Zr ions (r_Zr_ = 84 pm, r_Al_ = 67.5 pm) increase the stability of θ-Al_2_O_3_ and inhibit θ-Al_2_O_3_ (cubic) α-Al_2_O_3_ (hexagonal) transformation. Therefore, weight uptakes of Zr doped coating are bigger [33]. Then, from 20 h to 50 h, the weight of both coatings does not change significantly (up to 0.22 mg/cm^2^ for palladium doped coating and palladium and up to 0.45 mg/cm^2^ for palladium and zirconium co-doped coating). This may be attributed to the presence of protective oxide layers, formed in the initial stage of oxidation. In the further stage, the weight of the palladium doped coating is stable (up to 110 h) and then begins to fall. This suggests oxides’ spalling. At this time, the weight of the palladium and zirconium co-doped coating increases (up to 0.55 mg/cm^2^) and later begins to decrease (Figure 10b), but decreases slower (to 0.2 mg/cm^2^ after 250 h) than the palladium modified coating (to −0.03 mg/cm^2^ after 250 h^)^ (Figure 10a). It proves, that oxidation resistance of the palladium and zirconium co-doped aluminide coating is better than only palladium doped one. Therefore, it may be assumed that zirconium improves the oxidation resistance of Pd mono-doped aluminide coatings. A similar phenomenon was observed by Jiang et al. [30], who analyzed the influence of zirconium on the oxidation resistance of Zr + Pt co-doped aluminide coatings. Co-doped coatings have better oxidation resistance than Pt modified and not modified coatings. Zr incorporation improved spallation resistance and scale adherence. This behavior has been attributed to the fact that Zr atoms block Al diffusion. Diffusion paths of zirconium and aluminium are similar but not identical. Moreover, zirconium atoms diffuse slower than Al atoms (Zr diffusion activation energy is higher than Al diffusion activation energy). Therefore, zirconium blocks aluminum diffusion. Al_2_O_3_ oxide scale is formed through outward Al^+3^ cations and O^−2^ anions diffusion. As Al^+3^ cation diffusion is blocked by zirconium atoms, the oxide scale grows slower. During the oxidation process, voids are formed at the coating–oxide scale interface. The number of these voids depends on the surface energy of the coating–oxide scale interface. When small, separated voids grow and reach critical size, the coating begins to crack. Zirconium precipitates segregate in the middle and upper part of the additive zone, close to the coating/scale interface (Figure 3b and Figure 4a,b). These precipitates could prevent voids’ growth and coatings’ cracking and in this way improve scale adhesion [31].

## 4. Conclusions

- The Pd and Zr co-doped coating consists of two zones, the additive one (above) and the interdiffusion one (below).
- Both zones are built of the β–(Ni,Pd)Al phase, which proves that palladium has dissolved uniformly in the whole coating.
- A great number of TPC inclusions in the interdiffusion zone prevented crystallites coarsening
- Zirconium rich inclusions are situated in the additive zone, close to the coating’s surface
- The oxidation resistance of the palladium and zirconium co-doped aluminide coating is better than that of the palladium doped one. Zirconium could retard θ-Al_2_O_3_ to α-Al_2_O_3_ transformation and improve spallation resistance and scale adherence by blocking Al ions diffusion.

## Data Availability

The data presented in this study are available on request from the corresponding author.
